# The interplay of recombination landscape and a transposable element in European populations of *Chironomus riparius*

**DOI:** 10.1186/s12864-025-12130-7

**Published:** 2025-11-06

**Authors:** Laura Chiara Pettrich, Ann-Marie Waldvogel

**Affiliations:** 1 Institute of Zoology, Department of Biology, University of Cologne, Zülpicher Straße 47b, Cologne, 50674 Germany; 2 Limnological Research Station, School of Life Sciences, Technical University of Munich, Hofmark 1-3, Iffeldorf, 82393 Germany

**Keywords:** Population genomics, Chironomidae, Recombination variation, Transposable elements, Genome evolution

## Abstract

**Background:**

Broadening our taxonomic scope beyond model species offers deeper insights into the evolutionary dynamics of genomic processes such as recombination and the proliferation of transposable elements (TEs). TEs can drive substantial genomic rearrangements, yet the interplay between TEs and recombination remains poorly understood.

**Results:**

To investigate population-specific recombination patterns, we analysed the distribution of the species-specific *Cla*-element in the non-biting midge *Chironomus riparius*. This TE is known for its dynamic behaviour, exhibiting high numbers of unique insertions and population-specific distribution patterns. Its distribution showed no consistent association with recombination rates at the chromosome-wide scale. However, the *Cla*-element was often found outside haplotype blocks, suggesting it may be spatially separated from regions with low recombination.

**Conclusions:**

No strong association was found between the overall recombination landscape in *C. riparius* and the transposition activity of repetitive elements. Highlighting how the dynamics of transposable elements contribute to the complexity of genome evolution.

**Supplementary Information:**

The online version contains supplementary material available at 10.1186/s12864-025-12130-7.

## Background

Transposable elements (TEs) can change their position within the genome and play a significant role in genomic rearrangements [1], yet their broader impact on recombination landscapes remains largely unresolved [2]. TEs often accumulate in low-recombining or heterochromatic regions that are tightly packed, influenced by insertion bias and purifying selection against ectopic recombination [2]. Ectopic recombination is a crossing over between two homologous DNA sequences at non-allelic sites, typically deleterious, associated with genomic instability, and is distinct from the tightly regulated meiotic recombination [3–5]. However, some TEs may co-localize with recombination hotspots, suggesting they may also shape recombination variation [6]. Host organisms have evolved TE-silencing mechanisms, which could themselves modulate recombination landscapes [2, 7]. This complex interplay raises a fundamental question: do TEs influence recombination, or are recombination patterns shaped in response to TE activity and control [2]?

Recombination rates vary across chromosomes, typically with reduced rates in centromeres and heterochromatic regions, and elevated rates near telomeres [2, 8]. However, exceptions exist - for example, in plants, recombination rates are positively correlated with gene density, and hotspots may lie further from telomeres than expected [9]. Other factors influencing recombination distribution include centromere traits that vary between species, and holocentric centromeres may create a different pattern compared to localized centromeres. For example, in the holocentric wood white butterflies (*Leptidea sinapsis*), larger chromosomes show a bimodal recombination landscape, possibly due to interference between chiasmata [10]. Recombination rates were lower in subtelomeric regions, except in cases with chromosome rearrangements [10]. Similarly, mutation and recombination rates differ across species, populations, individuals, and even chromosomal regions [8, 11, 12]. Both processes have also been potentially linked to respond to environmental factors: mutation rates vary with ecological conditions [13], and experimentally evolved temperature-adapted *Drosophila melanogaster* populations exhibit higher recombination in warmer environments and lower rates in colder ones [14] which has also been observed in yeast species, with a roughly 1.2–1.3 fold change in average recombination rates due to temperatures [15]. However, experimentally obtained recombination rates might be different from natural conditions, causing variations based on experimental design and individuals tested [16].

Genome-wide patterns of TE distribution, recombination, and mutation - and their interactions - are thus critical for understanding evolutionary processes, including speciation, in both model and non-model species [17]. The increasing availability of high-quality reference genomes allows improved resolution of regions with variable recombination rates [8, 18] and those with high TE or repeat content [19]. These assemblies also enable the identification of haplotype blocks based on linkage disequilibrium (LD), where high LD is typically maintained within blocks, indicating that nearby variants are more often inherited together [20]. Longer haplotype blocks often indicate more recent recombination events and, if preserved, may reflect positive selection [21–23].

To investigate if TEs may influence recombination patterns across the genome or if the TEs are shaped by recombination, we focused on the non-biting midge *Chironomus riparius*, a non-model insect species with a well-characterized TE content and documented population structure [24]. Among its TEs, the *Cla*-element presents an especially informative candidate. We hypothesize that the recombination landscape of *C. riparius* is influenced by the distribution of the *Cla*-element across different genomic regions, and that this influence potentially leads to population-specific patterns of recombination. To test this, we analyzed genome-wide recombination patterns and haplotype block structure in relation to the distribution of *Cla*-element insertions across multiple populations.

This species-specific minisatellite-like transposable element is widely distributed across the genome [25, 26] and displays high levels of population-specific insertions, indicating dynamic activity [27]. It has been hypothesized to have spread as part of the speciation process within the *Chironomus* genus [26], making it an ideal case for studying TE-recombination interactions during evolutionary divergence.

The *Cla*-element is particularly intriguing due to its contrasting chromosomal distribution: it is restricted to centromeric regions in *Chironomus piger* but dispersed along chromosomal arms in *C. riparius* [28]. It comprises a 120 bp consensus sequence [25] and occurs both as monomeric repeats, which are short identical DNA motifs, and in large tandem-repetitive clusters, which are large, structured and organized arrays of such repeats [28, 29]. Its genome-wide insertion patterns vary among natural populations, and the detection of transcripts and heterozygous insertions supports the notion of ongoing transposition [27]. Additionally, it has been implicated in chromosomal breakpoints in heterochromatic regions [30], further supporting its potential role in shaping recombination variation [27].

## Materials and methods

### Data processing and variant calling

This study utilized the same datasets as [31]. We individually mapped the already trimmed Illumina reads of five European populations of *C. riparius* with four individuals each (ENA accession PRJEB24868) from Hesse in Germany (MG), Rhône-Alpes (MF) and Lorraine (NMF) in France, Piemont in Italy (SI) and Andalusia in Spain (SS), to the novel high-quality reference genome assembly (ENA accession PRJEB47883). All pre-processing and variant calling steps were performed according [31] and [32]. Reads were mapped with bwa mem (-M -R’@RG\tID:$Population\tSM:$Individual\tPL: ILLUMINA’, v0.7.17 [33]), to the reference genome. Filtering was performed using samtools (-q 30 -f 0 × 0002 -F 0 × 0004 -F0 × 0008, v1.13 [34]), and PicardTools (VALIDATIONSTRINGENCY SILENT -REMOVEDUPLICATES true, v2.26.10 [35]), to discard low quality alignments and duplicates. Following the MSMC2 workflow [36], variant calling and phasing were processed for the four chromosomes that were resolved. The unmasked reference genome was utilized to create mappability masks with SNPable [37]. With the software bcftools (v1.13 [38]), and the script bamCaller.py supplied by msmc-tools [39], variant calling was completed. Indels were ignored. SHAPEIT4 (v4.2 [40]), was used to perform phasing utilizing the previously generated VCF- and mask-files. With bcftools merge, we merged all VCF-files for phasing and separated them after completion. The phased and unphased VCF-files were merged to recover any missing information. During the phasing process, some sites might be omitted. By merging phased and unphased files, haplotype data is preserved; additionally, missing sites are supplemented, thereby increasing the number of informative sites. For further analysis, only biallelic sites were kept. Predicted centromere ranges from [31] were used using RepeatOBserver [41] with standard settings.

### Recombination landscape

To reconstruct the recombination landscape of *C. riparius*, we used the tool iSMC [42] and the provided scripts of [43]. This tool uses SMC models to infer population history and spatial variation in recombination rates. The artificial phased VCF-files of [31] (see “Data Availability” section) were used as input files. The chromosomes were analysed separately per individual. For each analysis, an iSMC option file needed to be provided, where we used the standard recommendations from the iSMC documentation (optimize = true, decode = true, decodebreakpointsparallel = false, numberrhocategories = 5, numberintervals = 40, functiontolerance = 1e-4, fragmentsize = 3000000). To obtain recombination rates (ρ) in windows for each individual, we used the iSMC mapper with a bin size of 1 kb, 10 kb, 100 kb, and 1 Mb. For each chromosome of each population, mean recombination rates (10 kb windows) were calculated based on the matching windows and then compared to the population-specific position of *Cla*-elements. Further details on all analysis steps can be found on GitHub (see “Data Availability” section).

### Transposable elements

The same repeat annotation as in [31] was used. Sensitive soft masking of repeats in the genome was conducted with RepeatMasker (v4.1.1 [44]), to detect repetitive regions and accurately annotate the *Cla*-element using a custom TE-library by Vladimir Kapitanov, complemented by TE-entries of [27]. Settings were a cutoff score 250 bp using the engine rmblast (-s -xsmall -cutoff 250 -u -gff -pa 10 -lib $LIB -dir $DIR -enginermblast $GENOME). The output file was converted to BED format using RM2Bed.py script supplied by RepeatMasker. Additionally, an annotation of the *Cla*-element population-wise was conducted using MELT (v2.2.2 [45]),. The necessary mei.zip file for the *Cla*-element was created following the instructions of the MELT documentation. To find the insertions, we added the MQ tag using samblaster to our sam-files and used the unfiltered but sorted bam-files as input for MELT-SPLIT. MELT-SPLIT runs in four different analysis steps, and data were obtained for each population and for all populations together (6 results in total). For the MELT run with all populations, we renamed insertions detected during the step where each individual is analyzed based on the following group analysis output using bedtools intersect (v2.31.0 [46]),. To visualise the insertions of the *Cla*-element in the genome of each population compared to their inferred recombination rates, R (v4.2.1 [47]), and R-Studio (v2022.02.0 + 433 [48]), was used together with several R packages, like tidyverse (v2.0.0 [49]), patchwork (v1.1.3 [50]), or cowplot (v1.1.1 [51]),. A detailed list of the R packages can be found in the Supplementary Table S1. Detected *Cla*-insertions were flagged unique when they only occurred in one population and flagged as shared when they occurred in more than one population.

To test whether the occurrence of *Cla* insertions is correlated to recombination events (100 kb windows), we used the closest function of bedtools, according to [43]. A larger window size was chosen to reduce potential bias in the analysis. R-Studio was used to create plots showing the recombination rate in relation to the nearest *Cla*-element. These decay plots were created using the distance of the next *Cla*-element to a recombination event. Based on the predicted centromere ranges, each chromosome was split into three parts and analysed separately. *Cla*-clusters with sizes smaller than 500 bp were investigated separately from *Cla*-clusters larger than or equal to 500 bp. The obtained measures were tested with the Pearson correlation coefficient using the R package stats, and the results were then visualised in a heatmap. Pearson correlation coefficients were calculated for each chromosome of each population to investigate whether the distance of the *Cla*-element is correlated to the amplitude of ρ. Smaller and larger clusters were again analysed independently from each other. ChatGPT was used for creating loops in R to improve data analysis and visualization scripts, as well as troubleshooting [52]. The suggestions from ChatGPT were carefully reviewed and confirmed for correctness.

### Haplotype blocks

To determine haplotype blocks, it was necessary to merge the variant calls of each chromosome and individual. We assigned unique variant identifiers (Chromosome: Position) using bcftools annotate (v1.19 [38]), to avoid conflicts in data formatting and merged chromosomes per individual using bcftools concat. We then corrected sample names using bcftools reheader. Individuals were merged within populations using bcftools merge, and unique variant identifiers were updated to the population level using bcftools annotate. SNPs only data was then merged across populations using again bcftools merge. This dataset was converted to PLINK format using PLINK (--vcf --make-bed --maf 0.10 --geno 0.05; v1.90b6.18 [53]),. Haplotype blocks were calculated using the same tool with the options: --blocks no-pheno-req no-small-max-span --blocks-max-kb 1000 --blocks-min-maf 0.10 --blocks-strong-lowci 0.65 --blocks-strong-highci 0.97 --blocks-inform-frac 0.90 --snps-only. The blocks.det file was loaded to RStudio, together with the MELT results of all populations, and were visualized together using the packages tidyverse, scales [54], reshape2 [55] and the BiocManager [56] packages GenomicRanges [57], rtracklayer [58], ggbio [59] and regioneR [60]. A Wilcoxon rank sum test was conducted for haplotype block length and *Cla*-element cluster size. An exact binomial test was used to determine whether *Cla*-elements are more likely to occur inside or outside haplotype blocks. Additionally, a permutation test was performed to assess how often *Cla*-elements are found outside haplotype blocks by chance, using 5000 permutations with fixed chromosomes, fixed length, and no overlap. To test whether the occurrence of haplotype blocks is correlated to recombination events (1 kb windows), we again used the closest function of bedtools, according to [43]. Decay plots were created using the distance of the next block to a recombination event. The obtained measures were tested with the Spearman’s Rank correlation coefficient using the R package stats and visualised in a heatmap.

## Results

### Cla-elements localize in high numbers outside haplotype blocks

**Fig. 1:**
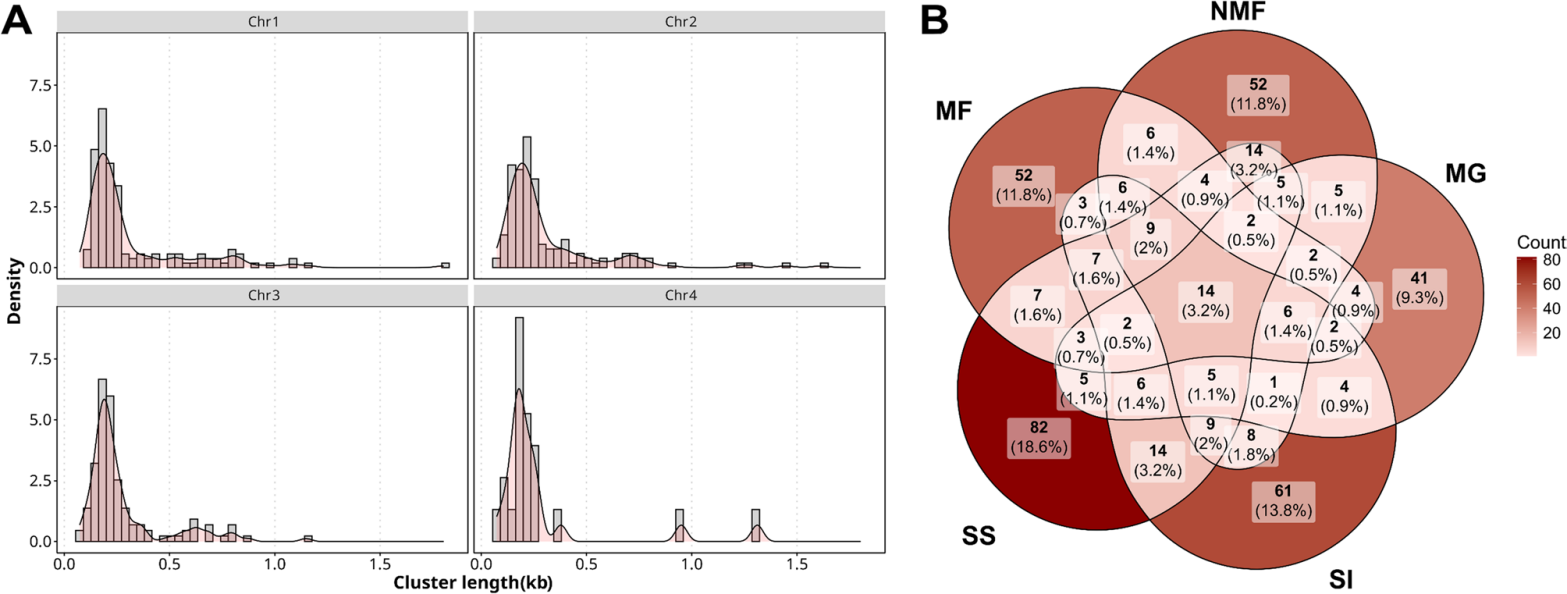
**A** Density plots showing the length frequency distribution of *Cla*-element clusters (in kb) for each chromosome integrated for all populations. **B** Venn diagram of unique and shared *Cla*-element clusters per population

The assembly includes 1492 monomeric repeats of the *Cla*-element, detected with RepeatMasker. Further genotyping with MELT revealed a total number of 441 tandem repetitive *Cla*-elements insertions (hereafter referred to as clusters) across all four chromosomes, integrating all populations, with eight clusters on the unplaced scaffolds. The lengths of the annotated *Cla*-elements varied between 73 bp and 1,802 bp with a mean of 304 bp (Fig. 1A). Most *Cla*-element clusters were counted on chromosome 1 with 148 clusters, followed by 143 on chromosome 2, 119 on chromosome 3, and the fewest on the shortest chromosome 4 with 20 clusters. Cluster counts per population resulted in a total number of 188 clusters across all chromosomes for the Andalusia (SS) population, 157 for the Piemont (SI) population, 148 for the Lorraine (NMF) population, 129 for the Rhône-Alpes (MF) population, and 107 for the Hesse (MG) population. Only 14 clusters were found to be shared among all populations, though 139 clusters occurred in two to four populations, all of which will be referred to as “shared clusters” (Fig. 1B). A total of 288 unique and thus population-specific *Cla*-clusters were detected, including the ones on the unplaced scaffolds (Fig. 1B, more details on the distribution of shared clusters in Supplementary Table S5). When looking at specific areas of the genome, we found in the centromere and flanking regions a total of 40 shared and 75 unique clusters. Considering chromosomal arms of chromosomes 1 to 3, 101 shared and 196 unique clusters were counted. The shortest chromosome 4 showed a total of 5 shared and 16 unique clusters. The unplaced scaffolds had 7 shared and 1 unique cluster (Supplementary Table S6).

**Fig. 2:**
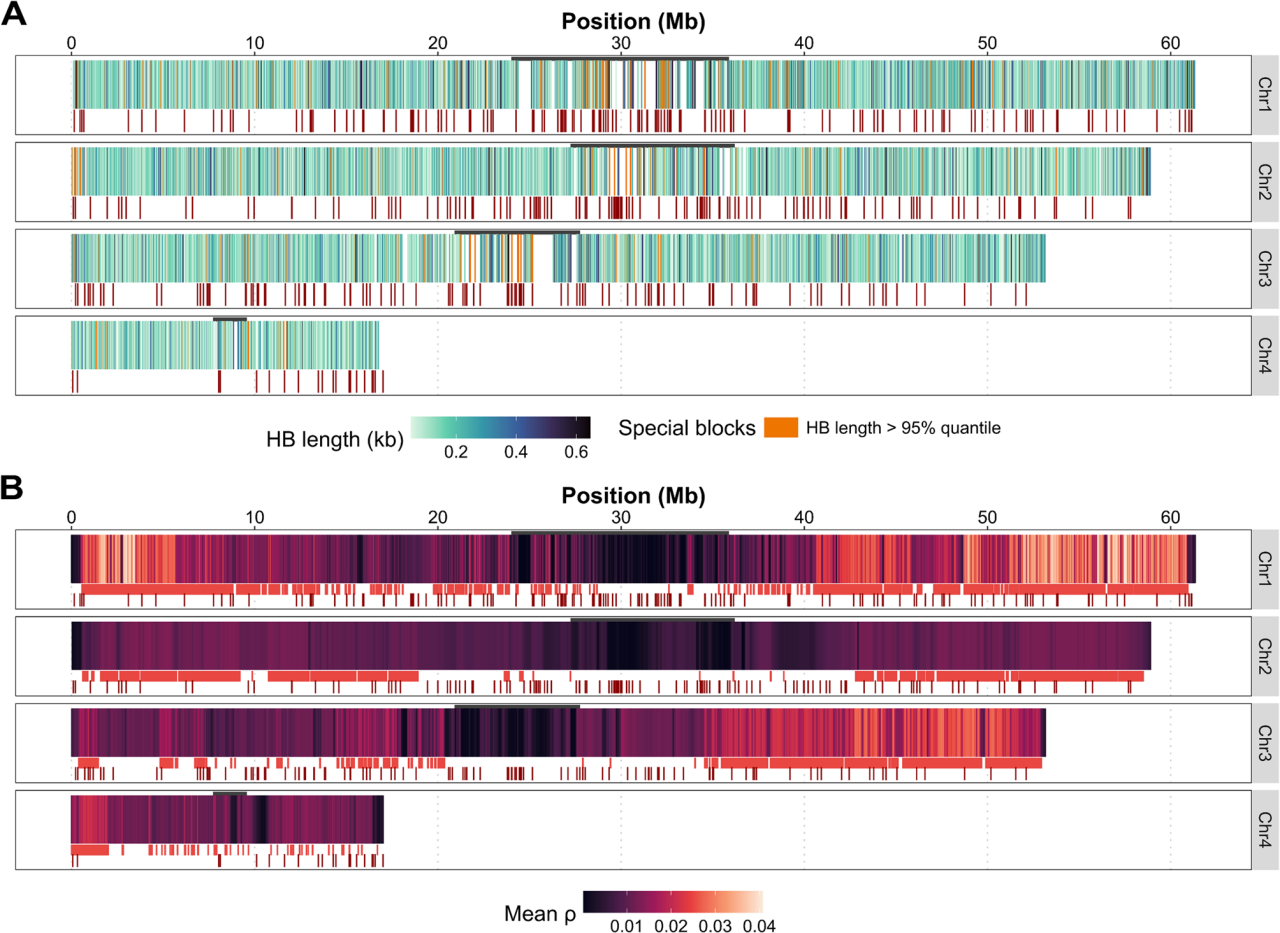
The genome-wide landscape of recombination and *Cla*-elements is illustrated through annotated heatmaps. Centromere regions are indicated at the top with a dark segment. The total number of *Cla*-element clusters, as annotated by MELT across all populations, is represented by dark red segments at the bottom of each plot. **A** Heatmap depicting determined haplotype blocks (kb). Haplotypes within the 95% quantile are highlighted in orange (>0.646 kb). **B** Heatmap illustrating mean ρ in 10 kb windows. Regions where ρ surpasses the chromosome mean by more than two standard deviations (mean + 2*SD) are classified as hotspots. Hotspots identified in any individual are displayed as bright red segments at the bottom. Only complete windows were included; values at window borders were excluded

We also compared the *Cla*-element distribution to patterns of genomic haplotype blocks. In total, 23,453 blocks were detected, ranging from 2 to 22,367 bp. To refine the data, we applied a minimum length threshold of 50 bp, excluding 9,848 shorter blocks and retaining 13,605 blocks with a mean length of 221 bp, which cover 1.58% of the genome. Most *Cla*-elements were located outside defined haplotype blocks. Excluding unplaced scaffolds, only 8 of 433 *Cla*-clusters overlapped with or fell within haplotype blocks (Fig. 2A). A Wilcoxon rank sum test showed this pattern was independent of *Cla*-element cluster size (*p* = 0.1377), but a second test indicated that intra-block clusters were significantly associated with longer blocks (*p* = 0.0001149; mean haplotype block length with *Cla*-cluster = 1,730 bp, without *Cla*-cluster = 220 bp). An exact binomial test further confirmed the bias toward clusters outside blocks (425 out of 433; *p* < 2.2e-16; success probability = 0.9815242).

Followed by this, we tested how likely it is that most of the *Cla*-elements are found outside a haplotype block using a permutation test (5000 permutations) with randomization of *Cla*-element positions and block positions (fixed chromosomes, fixed length, no overlap). Considering the actual genome structure, by random chance we find a permuted mean of 416.4 *Cla*-elements outside haplotype blocks with a permuted p-value of 0.01719656 (Z-score = -2.1518). The observed value (425 elements outside) is significantly higher than the random expectation, despite haplotype blocks representing only a small fraction of the genome.

### The genomic influence of the Cla-element on the recombination landscape

To explore the genome-wide recombination landscape in *C. riparius*, we estimated individual and population-specific recombination rates (ρ) in 10 kb windows across the genome. We found the highest mean ρ on chromosome 1 (ρ = 0.01573) and the lowest on chromosome 2 (ρ = 0.00909) (Table 1). Populations differed in recombination rates - the highest mean rates occurred in NMF (ρ = 0.02123), SI (ρ = 0.01469), and MF (ρ = 0.01071), and the lowest in SS (ρ = 0.00867) and MG (ρ = 0.00812).

**Table 1:** Global mean, median, and standard error of ρ from 10 kb windows for each chromosome and for each population (complete table of mean, median, and quartiles of ρ in supplementary table S3). N refers to the number of windows, i.e., recombination sites

Population	Chromosome	*N*	Mean ρ	Median ρ	SE low	SE high
total	Chr1	122,680	0.01573	0.00801	0.01564	0.01583
total	Chr2	117,740	0.00909	0.00909	0.00907	0.00911
total	Chr3	106,280	0.01338	0.01028	0.01331	0.01346
total	Chr4	34,020	0.01194	0.01299	0.01186	0.01203
MF	total	76,144	0.01071	0.00903	0.01065	0.01076
MG	total	76,144	0.00812	0.00891	0.00811	0.00814
NMF	total	76,144	0.02123	0.01071	0.02107	0.02139
SI	total	76,144	0.01469	0.00947	0.01461	0.01476
SS	total	76,144	0.00867	0.00941	0.00866	0.00869
total	total	380,720	0.01268	0.00930	0.01265	0.01272

**Fig. 3:**
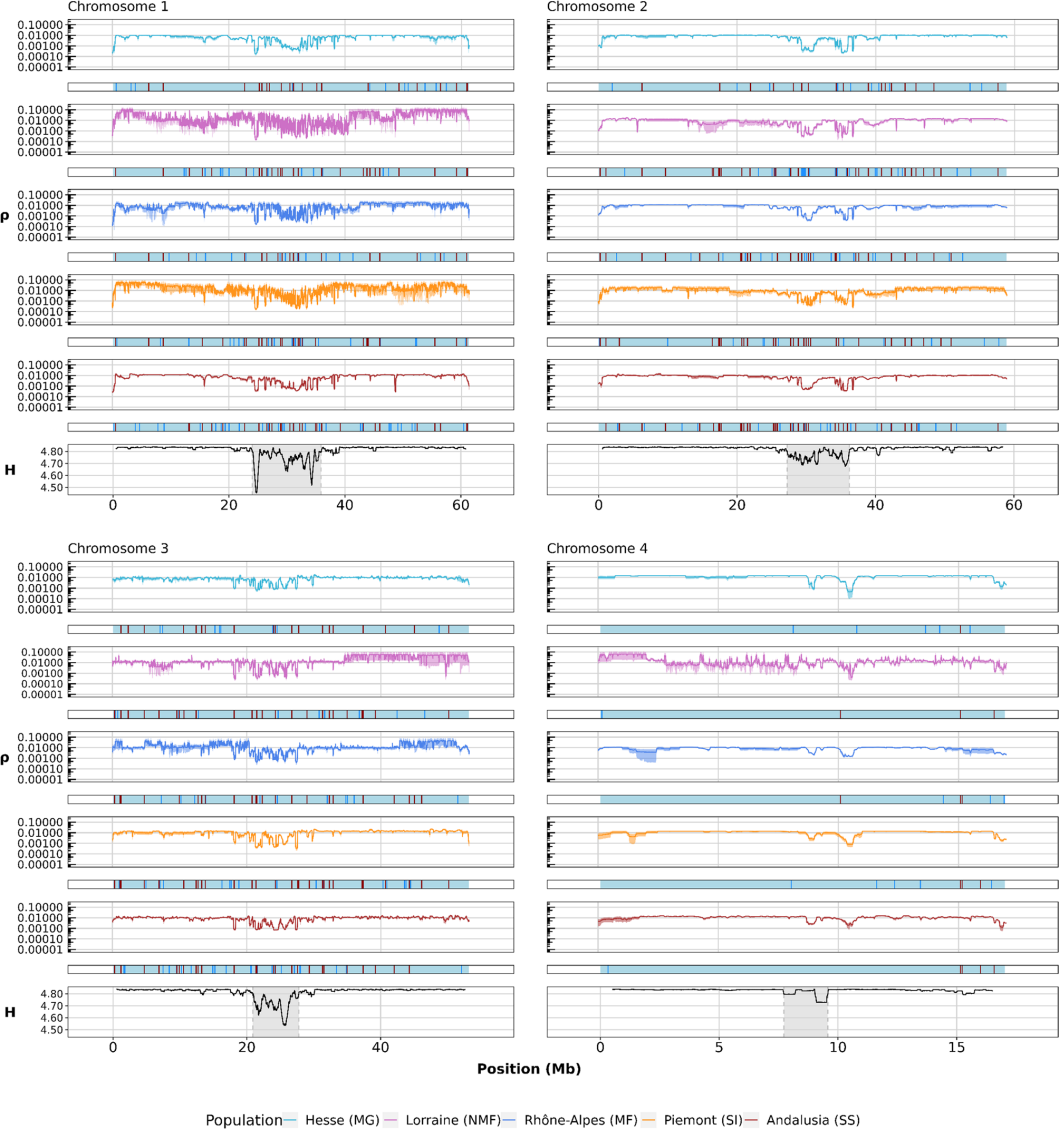
The mean recombination rate (ρ) (*n* = 4), with confidence intervals within 10 kb windows on a log10 scale, is compared to the position of the *Cla*-elements along each chromosome. The position is provided in megabases (Mb) at the bottom of each plot. Recombination rates and *Cla*-elements are depicted separately for each population and are color-coded accordingly. Dark red lines indicate the presence of a *Cla*-element found in multiple populations (i.e., shared). Unique inserts specific to one population are marked with bright blue lines. The variable ‘H’ refers to the Shannon diversity index estimated by RepeatObserver [41], representing the diversity of repeat lengths, with minimum values linked to the centromeres

The individual mean of ρ per chromosome decreased in areas near the centromeres and increased with greater distance to the centromeres (Fig. 2B). We used the global mean of ρ for each chromosome across individuals, along with twice its standard deviation (SD), to define hotspot regions (bright red segments at the bottom of Fig. 2B). The variation in ρ across chromosomes was found to be heterogeneous among populations, but major patterns appeared similar between populations (Fig. 3). Due to the smaller size of chromosome 4, with its unusual structures [61], and the uncertainty in centromere placement [31], we analyzed chromosome 4 in its entirety, without dividing it into separate arms and centromere regions.

**Fig. 4:**
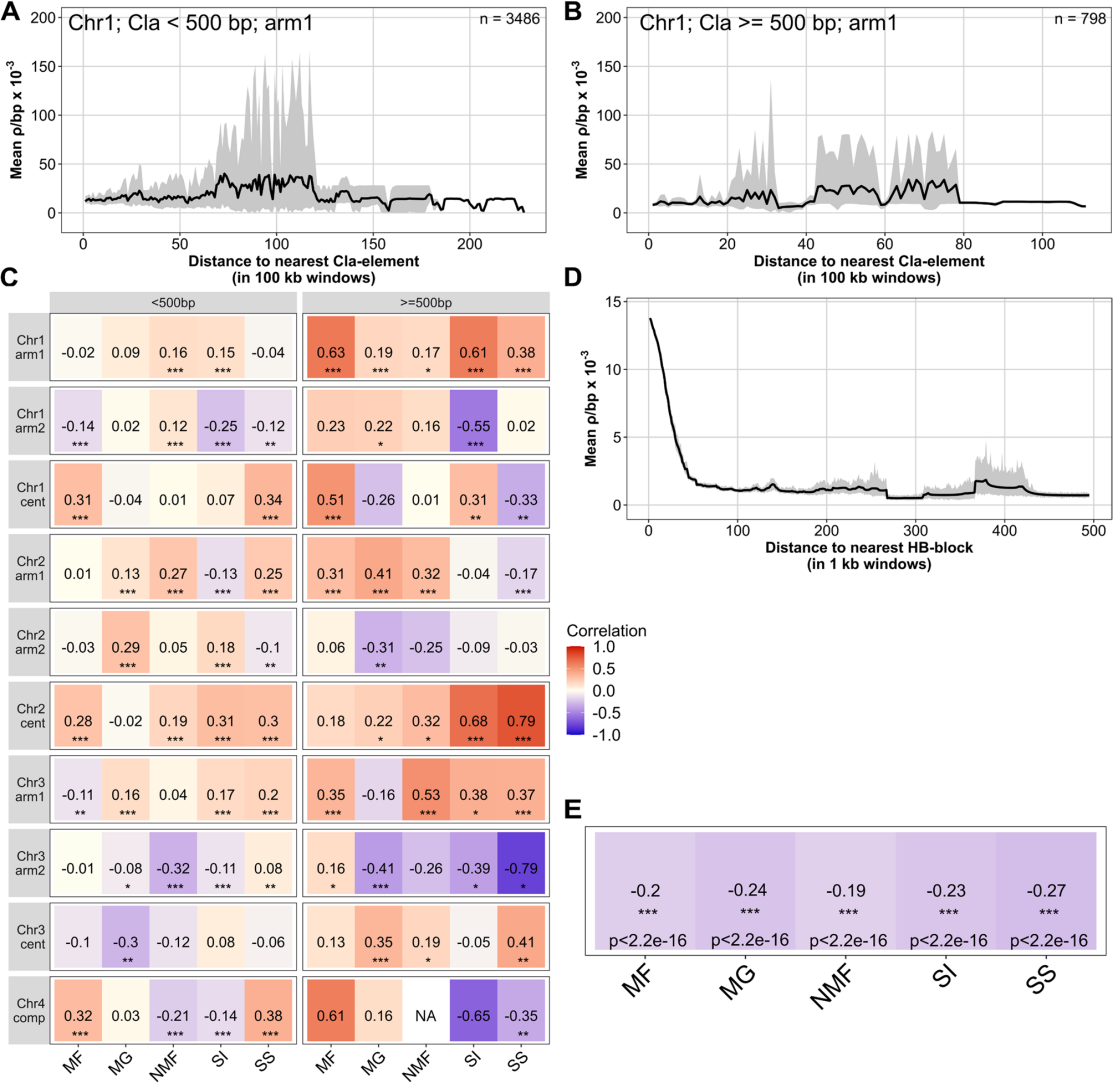
A-B) Mean recombination rate ρ per base pair in relation to the distance to the nearest *Cla*-element on chromosome 1, arm 1. Mean values were calculated for each window based on individual data (*n* = 20). Displayed in grey are 100 bootstrap values obtained through resampling the mean to visualize its distribution. **A** *Cla*-element cluster size less than 500 base pairs. **B** *Cla*-element cluster size equal to or greater than 500 base pairs. **C** Pearson correlation between the recombination rate ρ and the distance to the next *Cla*-element. An asterisk denotes statistical significance (* *p* < 0.05, ** *p* < 0.01, *** *p* < 0.001). A positive correlation indicates an increase in recombination rate as the distance to the *Cla*-element increases. The data for *Cla*-element cluster sizes less than 500 bp and those equal to or greater than 500 bp are presented. For these figures, recombination rates estimated in 100 kb windows were utilized; only complete windows were considered. For each chromosome, both arms and the centromere regions were analyzed separately; only chromosome 4 was analyzed completely. **D** Mean recombination rate ρ per base pair relative to the distance to the nearest haplotype block (HB). **E** Spearman correlation between the recombination rate ρ and the distance to the next HB. A negative correlation indicates a higher recombination rate with a closer HB. For decay analysis of HBs, recombination rates estimated in 1 kb windows were used; only complete windows were considered

To assess the relationship between *Cla*-elements and the recombination landscape, we analyzed the decay of ρ (estimated in 100 kb windows) with distance to the nearest *Cla*-element (Fig. 4A–C; Supplementary Figures S1–S10). We aimed to determine whether ρ increases (positive correlation) or decreases (negative correlation) with distance to the next *Cla*-element. The decay plots for the chromosomes were divided by cluster size of the *Cla*-element: smaller than 500 bp and larger than or equal to 500 bp (Fig. 4A-B). Correlation patterns varied across chromosomal arms and populations, with no consistent genome-wide trend (Fig. 4C, full statistics on Zenodo).

For small *Cla*-clusters (< 500 bp), 12 of 30 chromosomal arms showed low to moderate positive correlations (>0.125), while others showed negative correlations, including one strong case (>0.25) (Fig. 4C; Lorraine (NMF), Chr3, arm2). Larger clusters (= 500 bp) showed a similar but more pronounced pattern, with more frequent positive correlations, particularly on chromosome 1, yet still inconsistent overall (Fig. 4C).

Centromeric regions also showed mixed results (Fig. 4C). Positive correlations dominated for both small and large clusters, but strong negative correlations appeared in individual populations (e.g., Hesse (MG), Chr3 and Spain (SS), Chr1).

In summary, while positive correlations were more common - especially for larger clusters - substantial variation across chromosomes and populations did not reveal a clear or consistent relationship between *Cla*-elements and recombination. Correlations with unique or shared clusters were similarly inconclusive (Supplementary Figures S1–S6, full statistics on Zenodo).

Additionally, we also analysed the relationship between ρ and haplotype blocks (HB). HB boundaries are classified based on strong linkage disequilibrium (LD), and blocks are expected to be inherited more often together [53, 62]. We performed a decay analysis to investigate the amplitude of ρ in relation to the distance to the next haplotype block (Fig. 4D-E). We found a significant negative correlation in all populations, which indicates that ρ tends to be higher near haplotype blocks.

## Discussion

We characterized the genome-wide recombination landscape and examined the potential role of the *Cla*-element, a candidate transposable element linked to population divergence [27]. Analysing chromosomal arms and predicted centromere regions separately revealed a general tendency for *Cla*-elements to occur farther from regions of elevated recombination, although this pattern was not uniform across all genomic regions.

### Dynamics of the Cla-element

Recombination patterns, particularly in Lorraine (NMF), Rhône-Alpes (MF), and Piemont (SI), likely reflect the influence of genomic features such as microsatellites, transposable elements, telomeres, centromeres, and heterochromatin-rich regions, which shape overall genomic architecture [2, 27, 63, 64]. Among genomic markers, the transposable *Cla*-element is a strong candidate for explaining genome evolution in *C. riparius*, given its proposed role in speciation [28].

Previous studies found that 25% of *Cla*-clusters are shared across all populations [27]. Our results, based on a new reference genome, show an even lower proportion - 3.2% shared among all populations - with 34.7% present in at least two populations and 65.3% unique clusters. This high level of population-specific clusters suggests ongoing *Cla*-element activity. The Andalusia (SS) population, which shows the highest number of *Cla*-clusters, is geographically distant from Hesse (MG), which had the lowest number. This geographic differentiation aligns with previous reports of higher *Cla*-element activity and mutation rates in Andalusia [27, 65]. Migration analyses further suggest a European expansion originating from Rhône-Alpes (MF) [32], potentially influencing the distribution and diversity of *Cla*-elements. We also detected shorter *Cla*-elements than 120 bp, which may be explained by deletion or length heterogeneity within the oligo(dT) run documented for the *Cla*-element [26].

Most *Cla*-elements occur outside defined haplotype blocks, with only longer, presumably younger blocks containing clusters. This may be due to the repetitive nature of *Cla*-elements disrupting linkage or mechanisms, positioning them outside haplotype blocks. Although our genome assembly is of high resolution [31], it may still not fully capture the biological complexity, as evidenced by *Cla*-elements found on unplaced scaffolds. Individual characteristics of reference genomes, such as assembly completeness, structural variation, or unique repeat landscapes, can influence analyses of TE distribution or recombination. Tools like MELT [45] partly address this by identifying non-reference insertions that are missing from the reference assembly. Additionally, since haplotype block borders often coincide with recombination hotspots, *Cla*-elements could influence recombination in these regions in ways not detected by our analysis.

### Recombination rate ρ and its correlation to the next Cla-element

Advances in genome assembly now allow improved resolution of repetitive regions, providing new insights into genome dynamics and enabling estimation of population-specific recombination landscapes (Fig. 3). Regions of low recombination largely overlapped among populations, notably around centromeres, consistent with expectations [66]. Elevated recombination near haplotype block boundaries was also observed, aligning with their known association with historical recombination hotspots [20]. These improvements highlight the importance of high-quality assemblies for studying population genomics, especially in non-model organisms where such regions may evolve at different rates.

We expected *Cla*-elements to be associated with regions of low recombination; however, the data reveal a more complex pattern. After excluding short haplotype blocks, the remaining blocks encompass approximately 3 Mb, which constitutes roughly 1.58% of the genome. Therefore, it is not unexpected that the majority of *Cla*-elements are situated outside these regions. Among the small fraction of detected haplotype blocks, the *Cla*-element is observed more frequently outside the defined blocks than expected by chance. However, it is important to consider that the apparent separation of *Cla*-elements from haplotype blocks could be due to the limited genomic coverage of these blocks, rather than indicating a true insertion bias. Consequently, establishing a definitive connection between the location of *Cla*-elements and recombination rates remains challenging. Estimating haplotype blocks and their association with *Cla*-elements would be enhanced with an increased sample size; however, acquiring such samples may prove difficult when studying natural populations.

To explore potential links between recombination and *Cla*-element distribution in *C. riparius*, we compared recombination landscapes across four chromosomes (Figs. 3 and 4). Most populations showed a tendency for *Cla*-elements to occur farther from regions of elevated recombination, although no consistent global pattern emerged. This is also supported by our findings that most elements are located outside detected haplotype blocks, where we observed increased recombination rates at the boundaries of these blocks. TEs should disrupt the linkage between loci, causing haplotype blocks to be broken up. The large number of unique insertions in the population could also pose challenges in detecting haplotype blocks, as a larger sample size is needed to identify clear boundaries. One arm of chromosome 1 exhibited a clear positive correlation, indicating higher recombination rates with more distant *Cla*-elements, but correlations varied across chromosomes and populations. This heterogeneity persisted even after dividing chromosomes 1 to 3 into arms and centromeric regions to limit the scope of the analysis.

The performance of SMC-based models like iSMC may be affected by heterogeneous recombination patterns, potentially underestimating recombination rates [67]. *Cla*-element clusters vary in age; older clusters may have contributed to recombination suppression, while recent clusters may not yet exhibit linked effects [2]. We hypothesized that shared clusters are older than unique ones but found no clear evidence that shared clusters preferentially occur in low recombination regions (Supplementary Figures S1–S6). The high proportion of population-unique clusters supports ongoing transposition activity [27], which may obscure stable correlations. The *Cla*-element might be under selection against segregating sites [27], but the efficiency of selection might vary between high- and low-recombining regions, where in high-recombining regions, selection should be more effective due to reduced linkage (Hill–Robertson effect) [68]. This could explain more TEs in low-recombining regions, but insertion bias and demography might weaken or even reverse these effects. Over time, the *Cla*-element should be driven into heterochromatin-rich regions where it has been found to occur together with other elements of repetitive DNA [27]. Heterochromatic regions are areas with lower recombination [2], and along with a small effective population size, they can result in increased genetic drift and less effective selection, especially since the populations are thought to have split between the late Pleistocene and Holocene [31]. The distribution pattern of the *Cla*-element in different populations should also be highly influenced by the founders, leading to unique patterns that are shaped by genetic drift, selection, and migration between neighboring populations.

Initially, TEs were mainly studied in model organisms, with *Drosophila* having already more than forty years of research on this topic [7]. In *Drosophila*, the density of transposable elements (TEs) appears to be elevated in regions with low recombination rates. This correlation is likely due to the influence of purifying selection, which functions to eliminate harmful TE insertions that may induce ectopic recombination, disrupt gene integrity, or produce other deleterious effects. Additionally, the Hill-Robertson effect, which describes the interference between linked loci under selection, may also contribute to this phenomenon by reducing the efficiency of selection in low-recombining regions [7]. Together, these mechanisms might explain why TEs tend to accumulate in regions with low recombination rates. However, transposition activity is not constant over time, and some TEs are presumed to be highly dynamic, with some insertions potentially attributable to transposition bursts [7]. Recent TE activity in both *D. melanogaster* and *D. simulans* seems likely to be related to demographic history, with habitat expansion possibly prompting a burst of transposition across multiple TE families [69]. In *Arabidopsis lyrata*, demographic events may have shaped the number and frequency of TE insertions, contributing to potential TE accumulation. Purifying selection reduced TE frequency near genes, while some silenced TEs may have increased under positive selection, suggesting that both demography and selection influenced TE accumulation [70].

In summary, *Cla*-elements tend to be found more frequently in regions of low recombination, but no universal pattern was established. While correlations exist, causality remains unproven. The absence of a consistent pattern may reflect ongoing *Cla*-element activity or limited regulation of this transposable element [27]. If regulatory mechanisms influence *Cla*-element transposition, they could also affect local recombination rates. Notably, chromosomes with abundant *Cla*-elements show differential recombination patterns (Fig. 3), suggesting further comparative genomic analyses might clarify these relationships.

Our results highlight the complexity of genome evolutionary processes and suggest that the dynamic activity of transposable elements like the *Cla*-element may itself mask underlying genomic interaction patterns.

## Conclusion

We successfully characterized population-specific recombination landscapes, providing new insights into recombination variation across natural *C. riparius* populations. While our results did not reveal a consistent global association between *Cla*-element clusters and recombination rates, we observed a general tendency for *Cla*-elements to localize in regions of lower recombination, particularly near centromeres. This suggests that, despite ongoing transposition activity, the overall recombination landscape in *C. riparius* remains relatively stable concerning the impact of repetitive elements. Our findings highlight the complexity of genome evolution and highlight how the dynamic nature of transposable elements may obscure broader genomic interaction patterns, emphasizing the need for further detailed and comparative studies.

## Supplementary Information

Supplementary Material 1.

## Data Availability

At ENA you can find and download the genome assembly (accession PRJEB47883) and the trimmed Illumina sequences of the five populations, which were published under [32] (accession PRJEB24868). Variant calling files can be found in the Zenodo of [31]: 10.5281/zenodo.15177248. Scripts can be found in the GitHub repository https://github.com/lpettrich/Crip_RecombinationLandscape_HB_2025. Input files necessary to run the scripts will be available through Zenodo: 10.5281/zenodo.15536524.

## Abbreviations

HB: Haplotype blocks
LD: Linkage disequilibrium
MF: Rhône-Alpes in France
MG: Hesse in Germany
NMF: Lorraine in France
SD: Standard deviation
SI: Piemont in Italy
SS: Andalusia in Spain
TE: Transposable element
ρ: Population-specific recombination rates
