# Supplementary material for: The interplay of recombination landscape and a transposable element in European populations of Chironomus riparius
Source: BMC Genomics. 2025 Nov 6;26:1002. doi: 10.1186/s12864-025-12130-7 (PMC12593919; doi:10.1186/s12864-025-12130-7)
Supplement: Supplementary file 1 — Supplementary Material 1. [file 12864_2025_12130_MOESM1_ESM.pdf]

The interplay of recombination landscape and a transposable element in European populations of *Chironomus riparius*

Laura C. Pettrich [1] and Ann-Marie Waldvogel [1,2*]

Author details:

1 Institute of Zoology, University of Cologne, Cologne, Germany

2 Limnological Research Station, School of Life Sciences, Technical University of Munich, Hofmark 1-3, Iffeldorf 82393, Germany

*Authors for Correspondence:

Laura Chiara Pettrich, Institute of Zoology, University of Cologne, Cologne, Germany

and

Ann-Marie Waldvogel, Limnological Research Station, School of Life Sciences, Technical University of Munich, Munich, Germany

Supplementary Materials

Supplementary Table S1: List of tools and R packages used with citations.

	Tools	Version	Citation
**Software**	samtools	1.13	Li et al. (2009)
	bcftools	1.13	Li et al. (2009)
	bwa	0.7.17	Li (2013)
	Trimmomatic	0.39	Bolger et al. (2014)
	Picard Tools	2.26.10	Broad Institute (2018)
	FastQC	0.11.9	Andrews (2010)
	MultiQC	1.12	Ewels et al. (2016)
	Qualimap	2.2.2d	Okonechnikov et al. (2016)
	bedtools	2.31.0	Quinlan & Hall (2010)
	shapeit4	4.2	Delaneau et al. (2019)
	SNPable	-	Li (2009)
	iSMC	-	Barroso et al. (2019)
	RepeatMasker	4.1.1	Smit et al. (2015)
	RepeatOBserver	1	Elphinstone et al. (2025)
	MELT	2.2.2	Gardner et al. (2017)
	blastx	2.12.0	Camacho et al. (2009)
	BUSCO	5.3.2	Manni et al. (2021), Simão et al. (2015)
	BlobToolsKit	2.6.5	Challis et al. (2020)
	Rstudio	2022.02.0+433	RStudio Team (2020)
	R	4.2.1	R Core Team (2020)
**R packages**	ggplot2	3.3.6	Wickham (2016)
	dplyr	1.1.4	Wickham et al. (2023)
	tidyverse	2.0.0	Wickham et al. (2019)
	patchwork	1.1.3	Pedersen (2024)
	cowplot	1.1.1	Wilke (2024)
	scales	1.2.1	Wickham & Seidel (2022)
	RColorBrewer	1.1-3	Neuwirth (2022)
	reshape2	1.4.4	Wickham (2007)
	BiocManager	1.30.25	Morgan & Ramos (2025)
	GenomicRanges	1.58.0	Lawrence et al. (2013)
	rtracklayer	1.66.0	Lawrence et al. (2009)
	ggbio	1.54.0	Yin et al. (2012)
	regioneR	1.38.0	Gel et al. (2016)

Supplementary Results

Supplementary Table S2: Final diallelic SNP count per chromosome and individual of *Chironomus riparius* used as input in the models of iSMC.

Chromosme	Sample	SNP count
1	MF1	902,195
1	MF2	902,289
1	MF3	901,883
1	MF4	902,269
1	MG2	902,062
1	MG3	901,970
1	MG4	902,245
1	MG5	901,889
1	NMF1	901,953
1	NMF2	901,811
1	NMF3	902,160
1	NMF4	902,100
1	SI1	902,294
1	SI2	902,233
1	SI3	902,079
1	SI4	902,043
1	SS1	902,274
1	SS2	902,438
1	SS3	902,255
1	SS4	902,048
2	MF1	913,108
2	MF2	913,238
2	MF3	912,982
2	MF4	913,092
2	MG2	913,102
2	MG3	912,927
2	MG4	912,954
2	MG5	912,838
2	NMF1	913,117
2	NMF2	913,040
2	NMF3	912,912
2	NMF4	912,902
2	SI1	913,081
2	SI2	913,023
2	SI3	912,842
2	SI4	913,201
2	SS1	912,946
2	SS2	913,215
2	SS3	913,209
2	SS4	912,950
3	MF1	786,653
3	MF2	786,865
3	MF3	786,770
3	MF4	786,893
3	MG2	786,842

Chromosme	Sample	SNP count
3	MG3	786,771
3	MG4	786,804
3	MG5	786,641
3	NMF1	786,687
3	NMF2	786,703
3	NMF3	786,785
3	NMF4	786,815
3	SI1	787,059
3	SI2	787,032
3	SI3	786,868
3	SI4	786,907
3	SS1	786,861
3	SS2	787,109
3	SS3	786,921
3	SS4	786,701
4	MF1	246,169
4	MF2	246,126
4	MF3	246,121
4	MF4	246,198
4	MG2	246,135
4	MG3	246,171
4	MG4	246,137
4	MG5	246,187
4	NMF1	246,032
4	NMF2	246,125
4	NMF3	246,198
4	NMF4	246,130
4	SI1	246,229
4	SI2	246,200
4	SI3	246,234
4	SI4	246,218
4	SS1	246,184
4	SS2	246,201
4	SS3	246,175
4	SS4	246,138

Supplementary Table S3: Global mean, median and quartile (25 % and 75 %) of ρ for each chromosome of each population in 10 kb windows. Total values per population and chromosomes are also specified.

Population	Chromosome	N	Mean ρ	Median ρ	Q25	Q75	SE low	SE high
MF	Chr1	24536	0.00915354	0.00682537	0.00105129	0.01256492	0.00908653	0.00922055
MF	Chr2	23548	0.00845388	0.00985276	0.00546356	0.01212321	0.00842641	0.00848134
MF	Chr3	21256	0.01575148	0.01072447	0.00318987	0.01393899	0.01557013	0.01593283
MF	Chr4	6804	0.00833753	0.00917761	0.0053799	0.01313025	0.0082824	0.00839267
MG	Chr1	24536	0.00694967	0.00909468	0.00386812	0.00975813	0.00692573	0.0069736
MG	Chr2	23548	0.00827227	0.00906616	0.00703351	0.01207859	0.0082477	0.00829683
MG	Chr3	21256	0.00805335	0.00840476	0.00548901	0.01041494	0.00802291	0.00808378
MG	Chr4	6804	0.01204706	0.01306567	0.01290811	0.01470966	0.01199279	0.01210134
NMF	Chr1	24536	0.03253499	0.00878908	0.00094546	0.02820241	0.03215883	0.03291115
NMF	Chr2	23548	0.00923108	0.01070171	0.00251745	0.01501712	0.00919161	0.00927055
NMF	Chr3	21256	0.02270239	0.01408449	0.00278692	0.01655105	0.02240101	0.02300376
NMF	Chr4	6804	0.01738729	0.01254372	0.002574	0.01784705	0.01699122	0.01778336
SI	Chr1	24536	0.02170701	0.00795255	0.00175981	0.02248257	0.02149756	0.02191645
SI	Chr2	23548	0.01144847	0.00860399	0.00352192	0.0158034	0.01137648	0.01152046
SI	Chr3	21256	0.01129308	0.01324767	0.00780191	0.01609498	0.0112511	0.01133506
SI	Chr4	6804	0.01118637	0.0141794	0.00972672	0.01477015	0.01112667	0.01124606
SS	Chr1	24536	0.00832284	0.00931586	0.00497541	0.01301677	0.0082943	0.00835138
SS	Chr2	23548	0.00803629	0.00870265	0.00559995	0.01112717	0.0080133	0.00805928
SS	Chr3	21256	0.00911988	0.01024914	0.00704943	0.01052748	0.00909169	0.00914807
SS	Chr4	6804	0.01074792	0.01301527	0.00778858	0.01323771	0.01069637	0.01079948
total	Chr1	122680	0.01573361	0.0080094	0.00224922	0.01147715	0.01564166	0.01582555
total	Chr2	117740	0.0090884	0.0090861	0.00528871	0.0121025	0.00906947	0.00910732
total	Chr3	106280	0.01338404	0.01027503	0.00550595	0.01408882	0.01331084	0.01345723
total	Chr4	34020	0.01194123	0.01298938	0.00750376	0.0142109	0.01185742	0.01202505
MF	total	76144	0.0107061	0.00903036	0.00326805	0.01265381	0.01064904	0.01076316
MG	total	76144	0.00812227	0.00890807	0.00534283	0.01119422	0.00810688	0.00813767
NMF	total	76144	0.02122974	0.01070541	0.00187012	0.01653721	0.02107378	0.02138569
SI	total	76144	0.0146873	0.00947056	0.00385691	0.01585351	0.01461297	0.01476162
SS	total	76144	0.00867342	0.00940729	0.00565623	0.01126988	0.00865838	0.00868846
total	total	380720	0.01268377	0.00930122	0.00443159	0.01308853	0.01264629	0.01272124

Supplementary Table S4: Centromere prediction by (Pettrich et al. 2025).

**Chromosome**	**Length (bp)**	**Start (bp)**	**End (bp)**
Chr1	11,885,000	24,007,501	35,892,501
Chr2	8,970,000	27,232,501	36,202,501
Chr3	6,860,000	20,902,501	27,762,501
Chr4	1,850,000	7,722,501	9,572,501

Supplementary Table S5: Detailed information on number of unique and shared *Cla*-insertions per population.

**Population**	**Number of *Cla*-elements**
MF	52
MG	41
NMF	52
SI	61
SS	82
**Total number of unique insertions**	**288**
MF,MG	4
MF,MG,NMF	2
MF,MG,NMF,SI	6
MF,MG,NMF,SS	2
MF,MG,SI	2
MF,MG,SI,SS	2
MF,MG,SS	3
MF,NMF	6
MF,NMF,SI	6
MF,NMF,SI,SS	9
MF,NMF,SS	4
MF,SI	3
MF,SI,SS	7
MF,SS	7
MG,NMF	5
MG,NMF,SI	1
MG,NMF,SI,SS	5
MG,NMF,SS	5
MG,SI	4
MG,SI,SS	6
MG,SS	5
NMF,SI	8
NMF,SI,SS	9
NMF,SS	14
SI,SS	14
**Total number of shared insertions in subset of populations**	**139**
MF,MG,NMF,SI,SS	14
**Total number of shared insertions in all populations**	**14**

Supplementary Table S6: Information on number of unique and shared *Cla*-insertions per chromosome.

**Position**	**Presence**	**Number of *Cla*-elements**
Chr1 arm1	Shared	10
Chr1 arm1	Unique	38
Chr1 arm2	Shared	19
Chr1 arm2	Unique	34
Chr1 centromere	Shared	17
Chr1 centromere	Unique	30
**Total number of insertions on Chr1**		**148**
Chr2 arm1	Shared	21
Chr2 arm1	Unique	29
Chr2 arm2	Shared	17
Chr2 arm2	Unique	33
Chr2 centromere	Shared	15
Chr2 centromere	Unique	29
**Total number of insertions on Chr2**		**144**
Chr3 arm1	Shared	17
Chr3 arm1	Unique	36
Chr3 arm2	Shared	17
Chr3 arm2	Unique	26
Chr3 centromere	Shared	8
Chr3 centromere	Unique	16
**Total number of insertions on Chr3**		**120**
Chr4 complete	Shared	5
Chr4 complete	Unique	16
**Total number of insertions on Chr4**		**21**
Scaffold1 complete	Shared	1
**Total number of insertions on Scaffold1**		**1**
Scaffold3 complete	Shared	4
Scaffold3 complete	Unique	1
**Total number of insertions on Scaffold3**		**5**
Scaffold7 complete	Shared	2
**Total number of insertions on Scaffold7**		**2**

Supplementary Fig. S1: Mean recombination rate ρ/bp against distance to nearest *Cla*-element displayed for the MF population. Mean values were calculated for each window based on each individual (n = 4). Displayed in grey are 100 bootstrap values calculated by resampling the mean to visualise its distribution. Unique *Cla*-insertions of the population are displayed on the left and shared insertions are on the right. For these figures the recombination rates estimated in 100 kb windows were used, only complete windows were regarded.

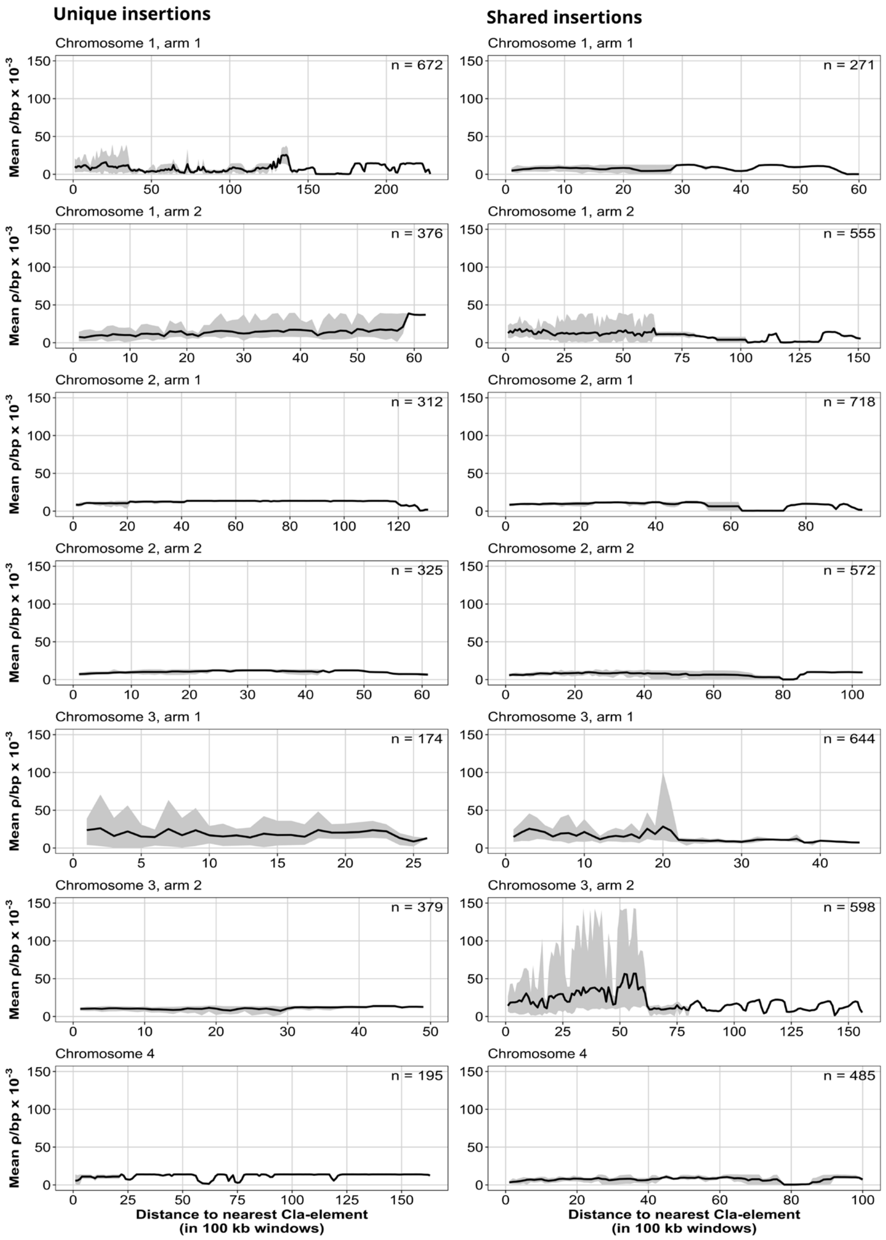

Supplementary Fig. S2: Mean recombination rate ρ/bp against distance to nearest *Cla*-element displayed for the MG population. Mean values were calculated for each window based on each individual (n = 4). Displayed in grey are 100 bootstrap values calculated by resampling the mean to visualise its distribution. Unique *Cla*-insertions of the population are displayed on the left and shared insertions are on the right. For these figures the recombination rates estimated in 100 kb windows were used, only complete windows were regarded.

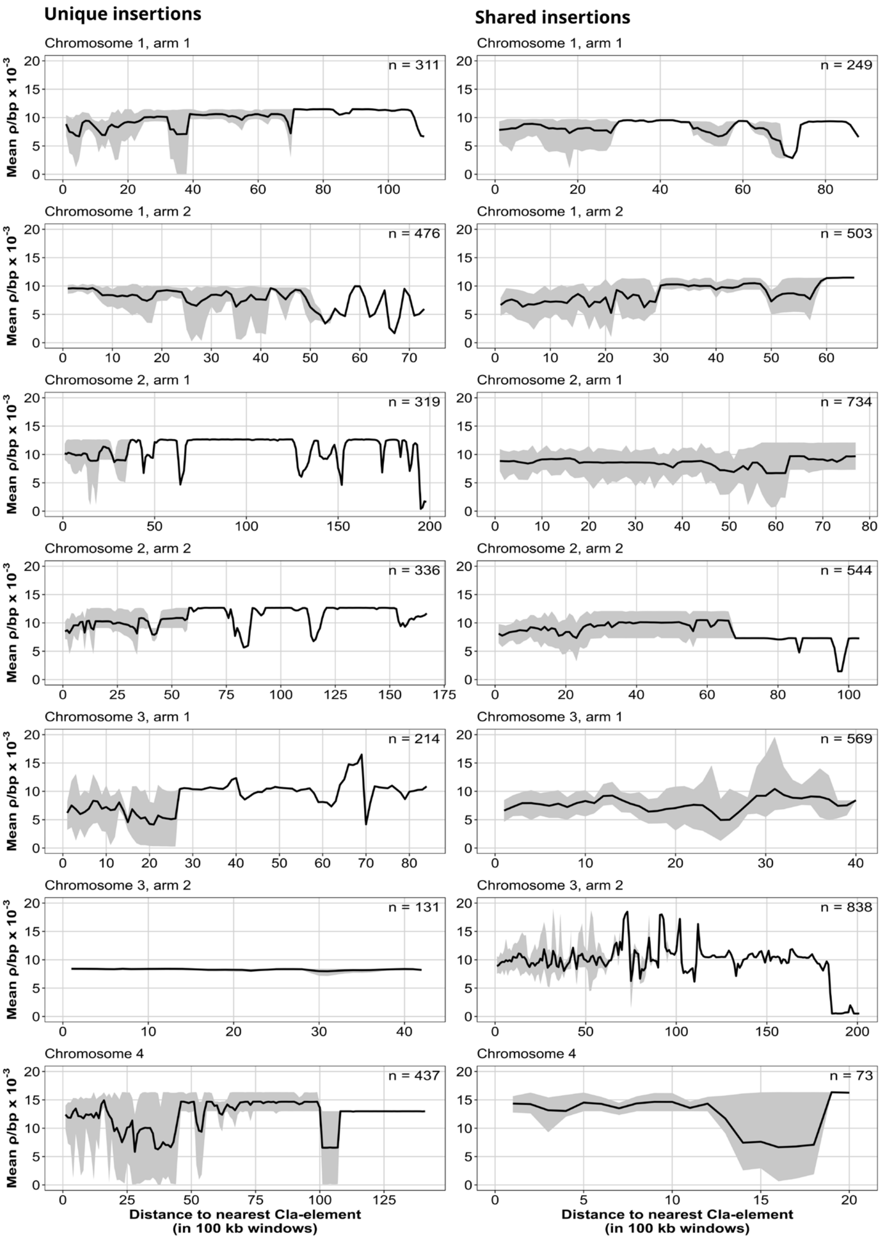

Supplementary Fig. S3: Mean recombination rate ρ/bp against distance to nearest *Cla*-element displayed for the NMF population. Mean values were calculated for each window based on each individual (n = 4). Displayed in grey are 100 bootstrap values calculated by resampling the mean to visualise its distribution. Unique *Cla*-insertions of the population are displayed on the left and shared insertions are on the right. For these figures the recombination rates estimated in 100 kb windows were used, only complete windows were regarded.

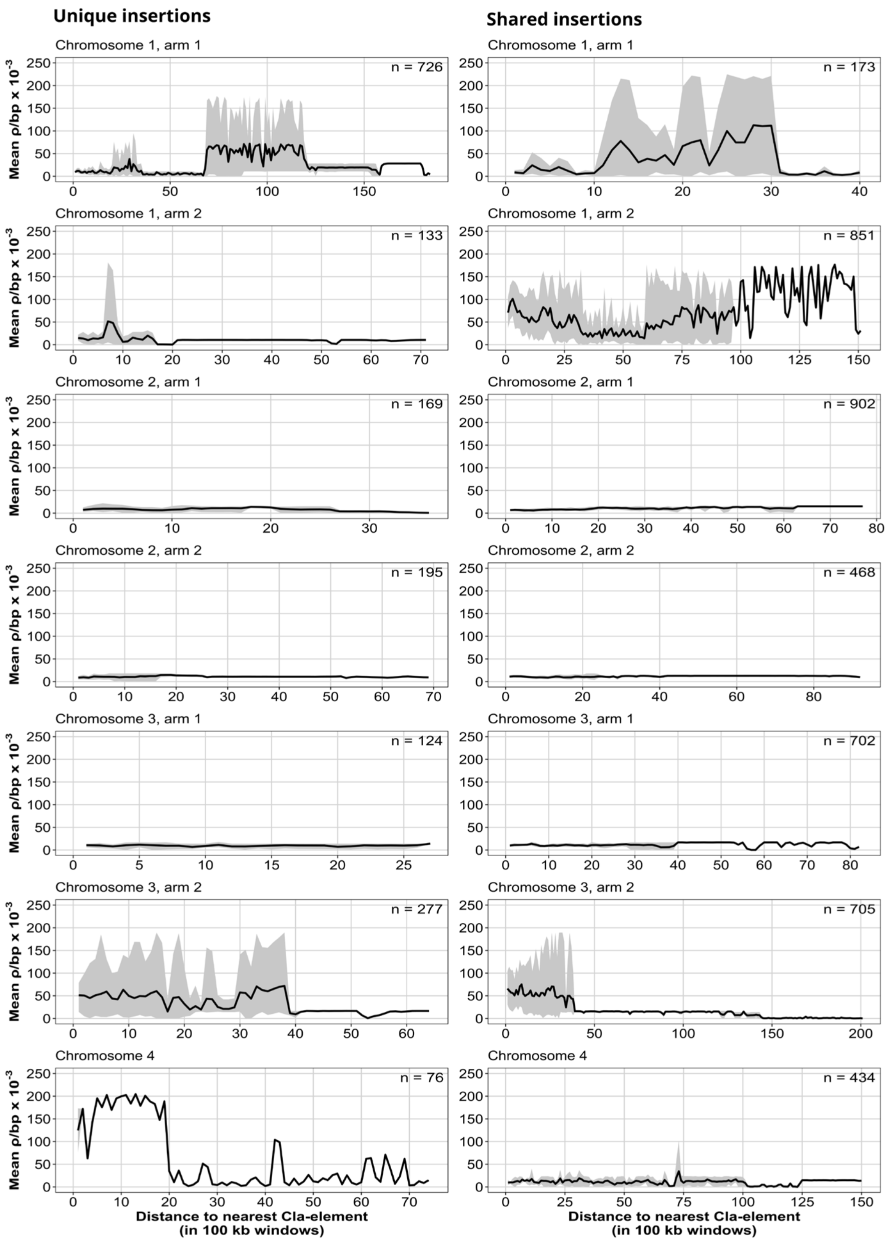

Supplementary Fig. S4: Mean recombination rate ρ/bp against distance to nearest *Cla*-element displayed for the SI population. Mean values were calculated for each window based on each individual (n = 4). Displayed in grey are 100 bootstrap values calculated by resampling the mean to visualise its distribution. Unique *Cla*-insertions of the population are displayed on the left and shared insertions are on the right. For these figures the recombination rates estimated in 100 kb windows were used, only complete windows were regarded.

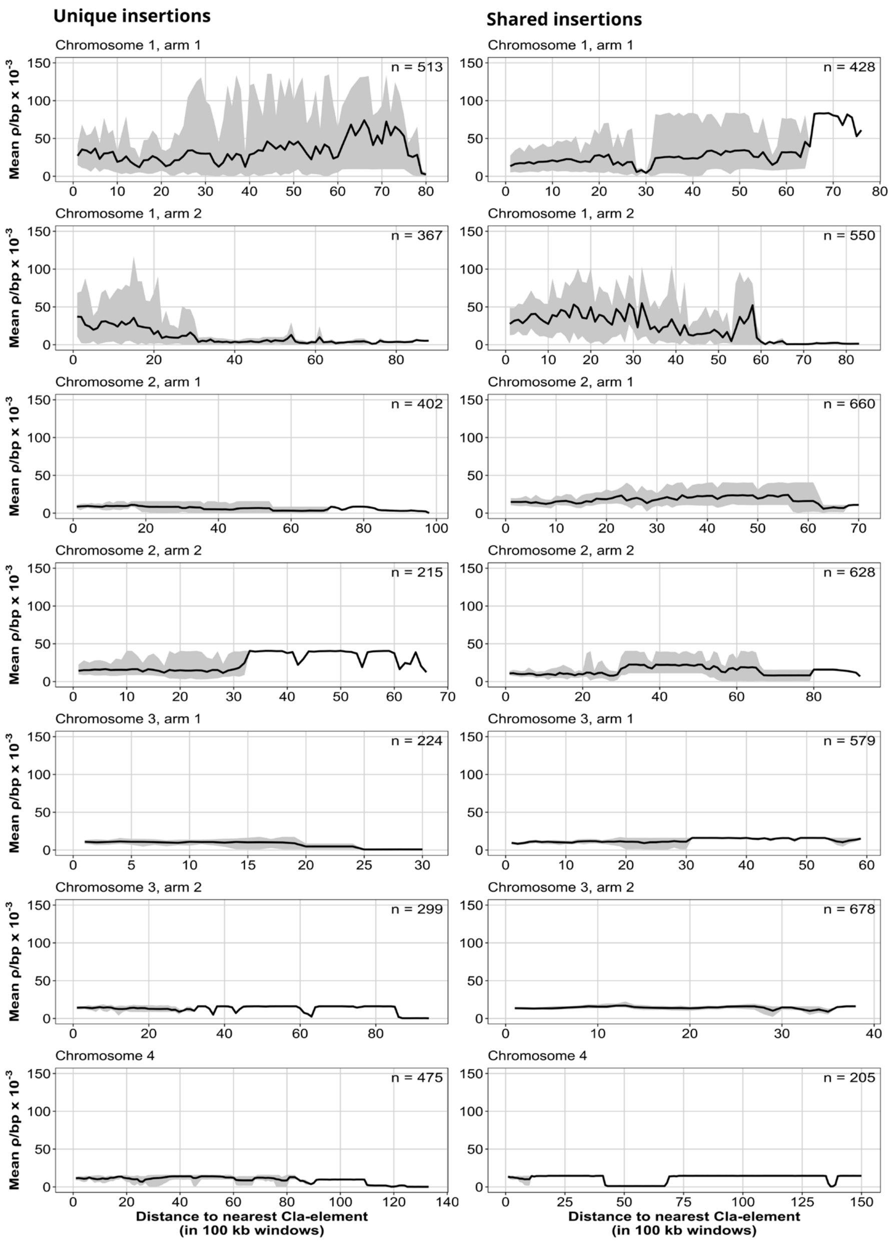

Supplementary Fig. S5: Mean recombination rate ρ/bp against distance to nearest *Cla*-element displayed for the SS population. Mean values were calculated for each window based on each individual (n = 4). Displayed in grey are 100 bootstrap values calculated by resampling the mean to visualise its distribution. Unique *Cla*-insertions of the population are displayed on the left and shared insertions are on the right. For these figures the recombination rates estimated in 100 kb windows were used, only complete windows were regarded.

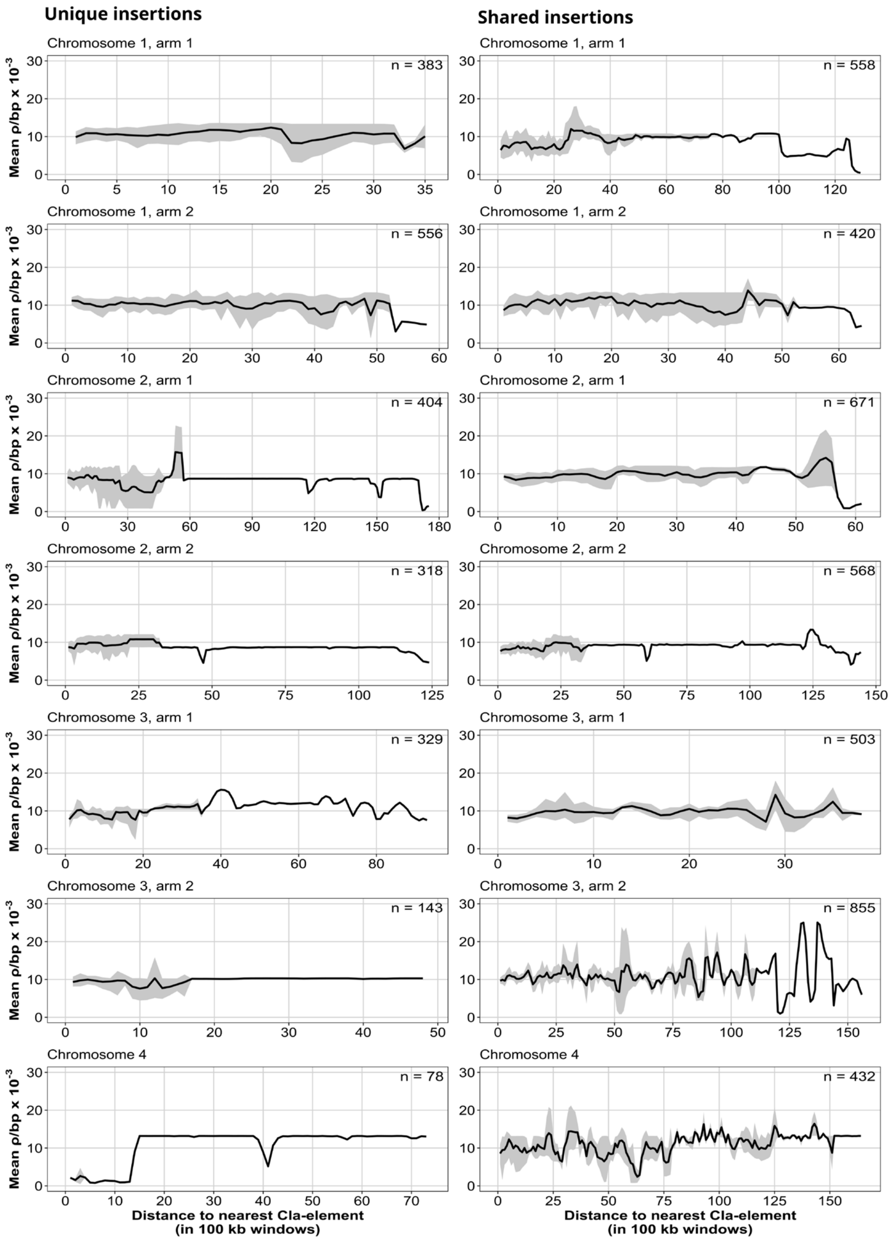

Supplementary Fig. S6: Pearson correlation between recombination rate ρ and distance to next *Cla*-element. An asterisk indicates significance (* p < 0.05, ** p < 0.01, *** p < 0.001). Positive correlation indicates a higher recombination rate with a more distant *Cla*-element. Unique *Cla*-elements of one population and *Cla*-elements shared in at least two populations are displayed. For these figures the recombination rates estimated in 100 kb windows were used, only complete windows were regarded.

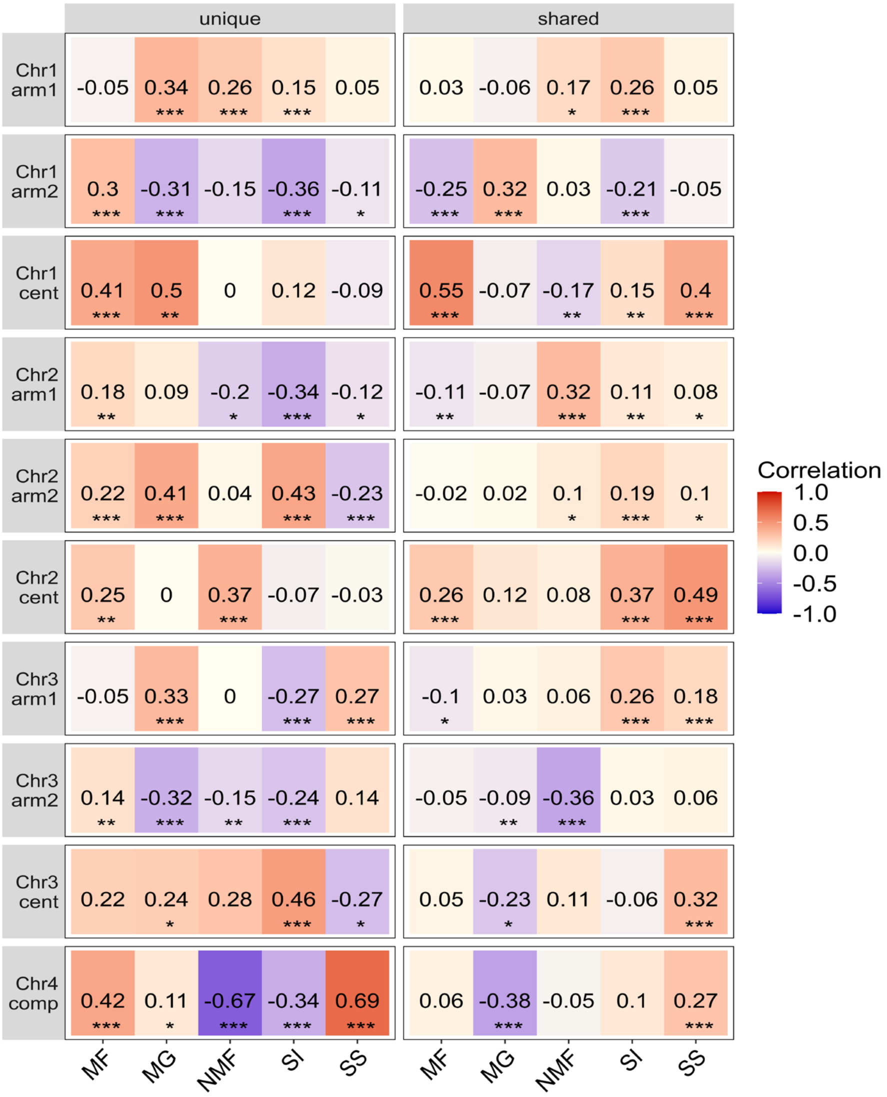

Supplementary Fig. S7: Mean recombination rate ρ/bp against distance to nearest *Cla*-element. Mean values were calculated for each window based on each individual (n = 20). Displayed in grey are 100 bootstrap values calculated by resampling the mean to visualise its distribution. *Cla*-element cluster size <500 bp.

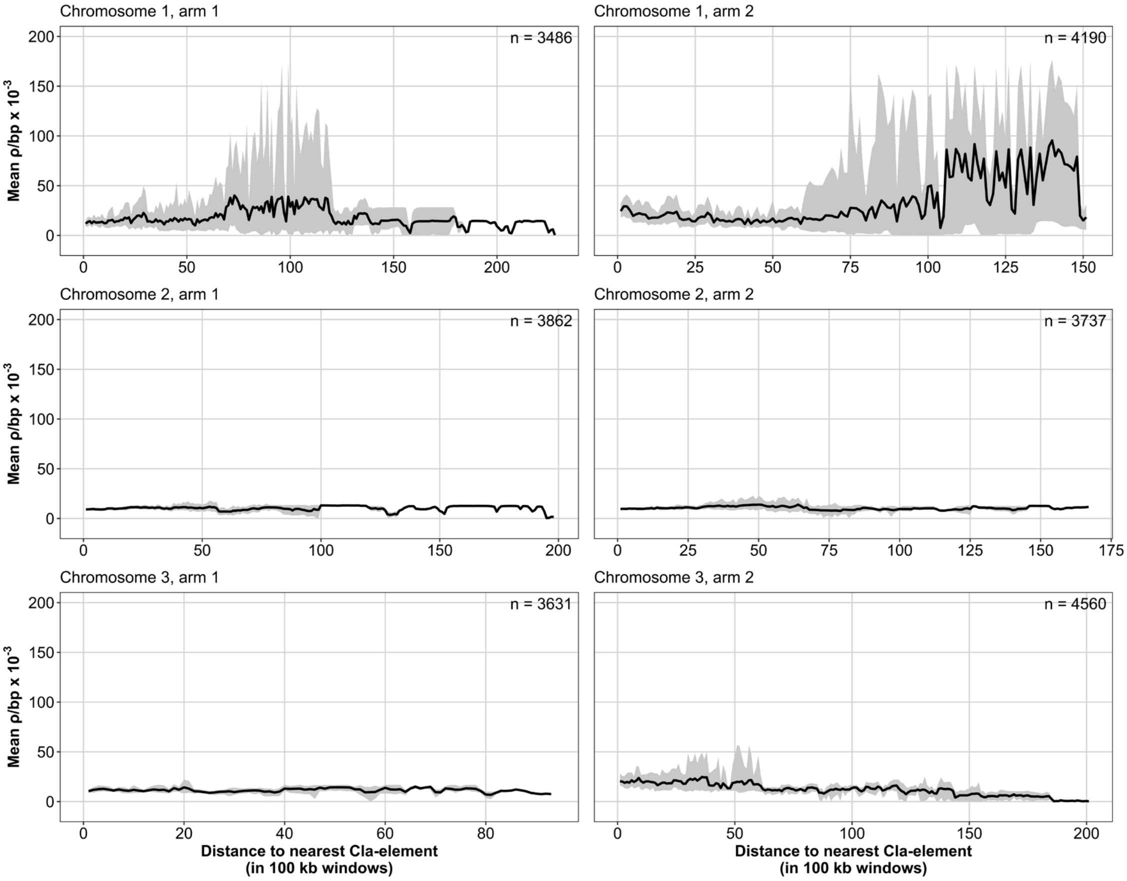

Supplementary Fig. S8: Mean recombination rate ρ/bp against distance to nearest *Cla*-element. Mean values were calculated for each window based on each individual (n = 20). Displayed in grey are 100 bootstrap values calculated by resampling the mean to visualise its distribution. *Cla*-element cluster size >=500 bp.

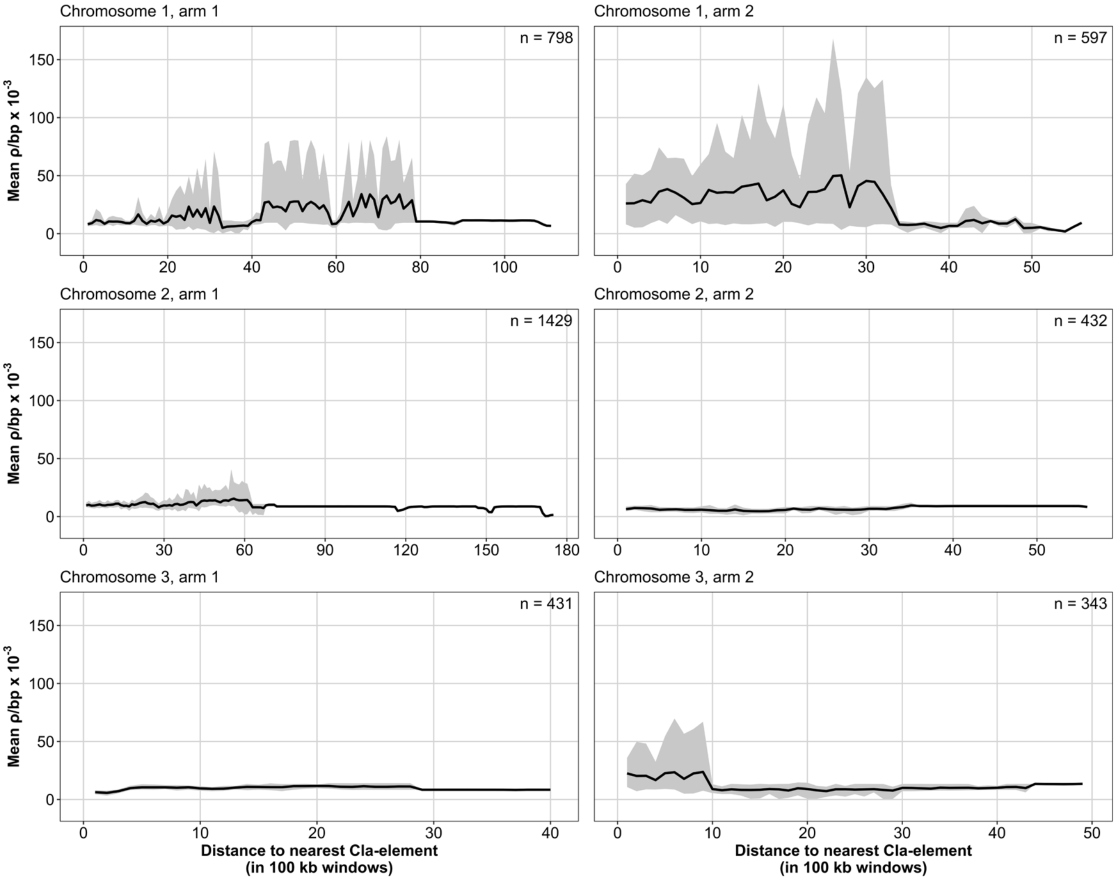

Supplementary Fig. S9: Mean recombination rate ρ/bp against distance to nearest *Cla*-element of centromere region. Mean values were calculated for each window based on each individual (n = 20). Displayed in grey are 100 bootstrap values calculated by resampling the mean to visualise its distribution. *Cla*-element cluster size <500 bp.

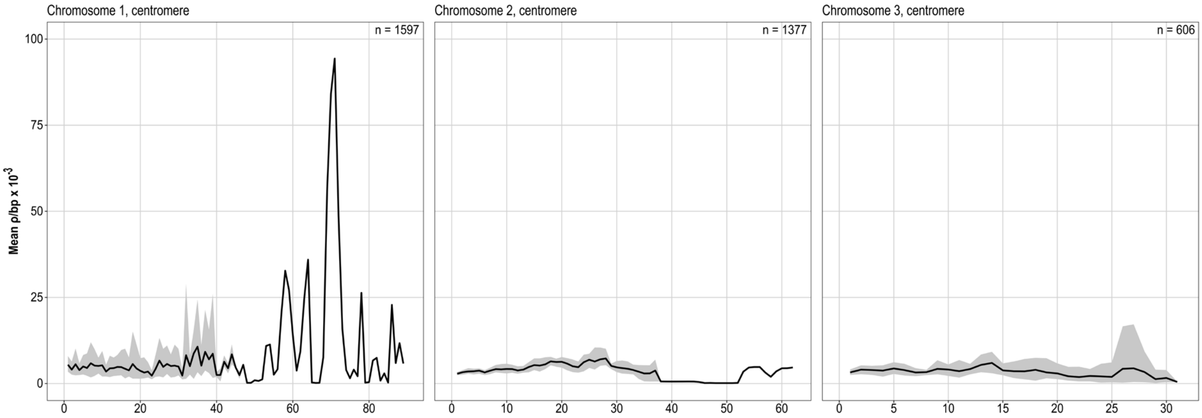

Supplementary Fig. S10: Mean recombination rate ρ/bp against distance to nearest *Cla*-element of centromere region. Mean values were calculated for each window based on each individual (n = 20). Displayed in grey are 100 bootstrap values calculated by resampling the mean to visualise its distribution. *Cla*-element cluster size >=500 bp.

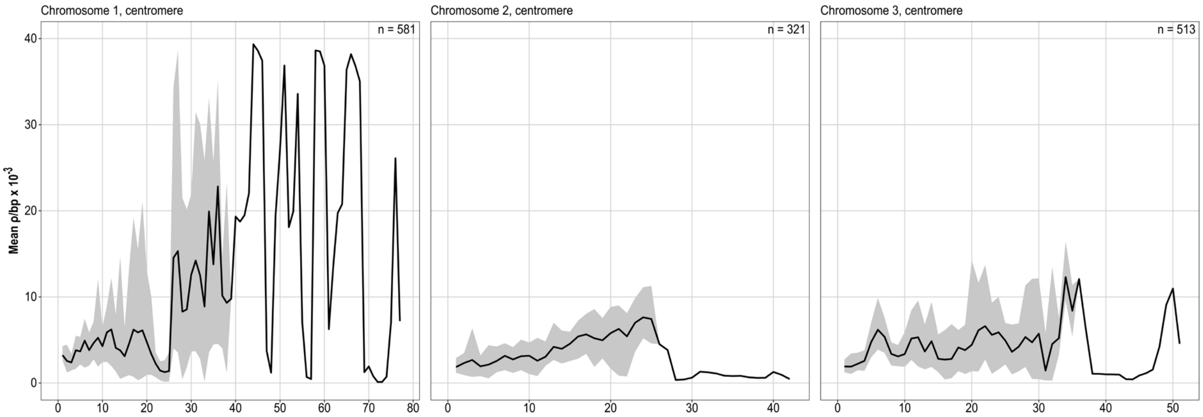

References

Andrews S. 2010. FastQC: A Quality Control Tool for High Throughput Sequence Data. Babraham Bioinformatics. http://www.bioinformatics.babraham.ac.uk/projects/fastqc/.

Barroso G V., Puzović N, Dutheil JY. 2019. Inference of recombination maps from a single pair of genomes and its application to ancient samples. PLoS Genet. 15:e1008449. doi: 10.1371/JOURNAL.PGEN.1008449.

Bolger AM, Lohse M, Usadel B. 2014. Trimmomatic: a flexible trimmer for Illumina sequence data. Bioinformatics. 30:2114–2120. doi: 10.1093/BIOINFORMATICS/BTU170.

Broad Institute. 2018. Picard Tools. http://broadinstitute.github.io/picard/.

Camacho C et al. 2009. BLAST+: Architecture and applications. BMC Bioinformatics. 10:1–9. doi: 10.1186/1471-2105-10-421.

Challis R, Richards E, Rajan J, Cochrane G, Blaxter M. 2020. BlobToolKit – Interactive Quality Assessment of Genome Assemblies. G3 Genes|Genomes|Genetics. 10:1361–1374. doi: 10.1534/G3.119.400908.

Delaneau O, Zagury JF, Robinson MR, Marchini JL, Dermitzakis ET. 2019. Accurate, scalable and integrative haplotype estimation. Nature Communications 2019 10:1. 10:1–10. doi: 10.1038/s41467-019-13225-y.

Elphinstone C, Elphinstone R, Todesco M, Rieseberg LH. 2025. RepeatOBserver: Tandem Repeat Visualisation and Putative Centromere Detection. Mol Ecol Resour. e14084. doi: 10.1111/1755-0998.14084.

Ewels P, Magnusson M, Lundin S, Käller M. 2016. MultiQC: Summarize analysis results for multiple tools and samples in a single report. Bioinformatics. 32:3047–3048. doi: 10.1093/bioinformatics/btw354.

Gardner EJ et al. 2017. The Mobile Element Locator Tool (MELT): population-scale mobile element discovery and biology. Genome Res. 27:1916–1929. doi: 10.1101/GR.218032.116.

Gel B et al. 2016. regioneR: an R/Bioconductor package for the association analysis of genomic regions based on permutation tests. Bioinformatics. 32:289–291. doi: 10.1093/BIOINFORMATICS/BTV562.

Lawrence M et al. 2013. Software for Computing and Annotating Genomic Ranges. PLoS Comput Biol. 9:e1003118. doi: 10.1371/JOURNAL.PCBI.1003118.

Lawrence M, Gentleman R, Carey V. 2009. rtracklayer: an R package for interfacing with genome browsers. Bioinformatics. 25:1841–1842. doi: 10.1093/BIOINFORMATICS/BTP328.

Li H. 2013. Aligning sequence reads, clone sequences and assembly contigs with BWA-MEM. doi: 10.48550/ARXIV.1303.3997.

Li H. 2009. SNPable Regions. http://lh3lh3.users.sourceforge.net/snpable.shtml.

Li H et al. 2009. The Sequence Alignment/Map format and SAMtools. Bioinformatics. 25:2078–2079. doi: 10.1093/bioinformatics/btp352.

Manni M, Berkeley MR, Seppey M, Zdobnov EM. 2021. BUSCO: Assessing Genomic Data Quality and Beyond. Curr Protoc. 1:e323. doi: 10.1002/CPZ1.323.

Morgan M, Ramos M. 2024. BiocManager: Access the Bioconductor Project Package Repository. https://CRAN.R-project.org/package=BiocManager.

Neuwirth E. 2022. RColorBrewer: ColorBrewer Palettes. R package version 1.1-3. https://CRAN.R-project.org/package=RColorBrewer (Accessed March 18, 2024).

Okonechnikov K, Conesa A, García-Alcalde F. 2016. Qualimap 2: advanced multi-sample quality control for high-throughput sequencing data. Bioinformatics. 32:292–294. doi: 10.1093/bioinformatics/btv566.

Pedersen TL. 2024. patchwork: The Composer of Plots. https://patchwork.data-imaginist.com.

Pettrich LC, King R, Field LM, Waldvogel A-M. 2025. High quality genome assembly of *Chironomus riparius* and its population history in European populations. G3: Genes|Genomes|Genetics. doi: 10.1093/g3journal/jkaf189.

Quinlan AR, Hall IM. 2010. BEDTools: a flexible suite of utilities for comparing genomic features. Bioinformatics. 26:841–842. doi: 10.1093/BIOINFORMATICS/BTQ033.

R Core Team. 2020. R: A language and environment for statistical computing. https://www.R-project.org/.

RStudio Team. 2020. RStudio: Integrated Development Environment for R. https://www.rstudio.com/.

Simão FA, Waterhouse RM, Ioannidis P, Kriventseva E V., Zdobnov EM. 2015. BUSCO: assessing genome assembly and annotation completeness with single-copy orthologs. Bioinformatics. 31:3210–3212. doi: 10.1093/BIOINFORMATICS/BTV351.

Smit AFA, Hubley R, Grenn P. 2015. RepeatMasker Open-4.0. http://www.repeatmasker.org.

Wickham H. 2016. ggplot2: Elegant Graphics for Data Analysis. https://ggplot2.tidyverse.org.

Wickham H. 2007. Reshaping Data with the reshape Package. J Stat Softw. 21:1–20. doi: 10.18637/JSS.V021.I12.

Wickham H et al. 2019. Welcome to the Tidyverse. J Open Source Softw. 4:1686. doi: 10.21105/JOSS.01686.

Wickham H, François R, Henry L, Müller K, Vaughan D. 2023. dplyr: A Grammar of Data Manipulation.

Wickham H, Seidel D. 2022. scales: Scale Functions for Visualization. R package version 1.2.1. https://CRAN.R-project.org/package=scales.

Wilke CO. 2024. cowplot: Streamlined Plot Theme and Plot Annotations for ‘ggplot2’. https://CRAN.R-project.org/package=cowplot.

Yin T, Cook D, Lawrence M. 2012. ggbio: an R package for extending the grammar of graphics for genomic data. Genome Biol. 13:R77. doi: 10.1186/GB-2012-13-8-R77/FIGURES/10.
